# Digital Delivery of Meditative Movement Training Improved Health of Cigarette-Smoke-Exposed Subjects

**DOI:** 10.3389/fpubh.2018.00282

**Published:** 2018-10-19

**Authors:** Peter Payne, Steven Fiering, David Zava, Thomas J. Gould, Anthony Brown, Paul Hage, Carole Gaudet, Mardi Crane-Godreau

**Affiliations:** ^1^Department of Microbiology and Immunology, Geisel School of Medicine at Dartmouth, Lebanon, PA, United States; ^2^ZRT Laboratory, Beaverton, OR, United States; ^3^Department of Biobehavioral Health, Pennsylvania State University, University Park, PA, United States; ^4^Roswell Park Comprehensive Cancer Center, Buffalo, NY, United States

**Keywords:** meditative movement, COPD, Qigong, flight attendants, digital training, interoception, video training, autonomic nervous system

## Abstract

Many FA who flew prior to the ban on smoking in commercial aircraft exhibit an unusual pattern of long-term pulmonary dysfunction. This randomized controlled study tested the hypothesis that digitally delivered meditative movement (MM) training improves chronic obstructive pulmonary disease (COPD)-related symptoms in flight attendants (FA) who were exposed to second-hand cigarette smoke (SHCS) while flying. Phase I of this two-phase clinical trial was a single-arm non-randomized pilot study that developed and tested methods for MM intervention; we now report on Phase II, a randomized controlled trial comparing MM to a control group of similar FA receiving health education (HE) videos. Primary outcomes were the 6-min walk test and blood levels of high sensitivity C-reactive protein (hs-CRP). Pulmonary, cardiovascular, autonomic and affective measures were also taken. There were significant improvements in the 6-min walk test, the Multidimensional Assessment of Interoceptive Awareness (MAIA) score, and the COPD Assessment Test. Non-significant trends were observed for increased dehydroepiandrosterone sulfate (DHEAS) levels, decreased anxiety scores and reduced blood hs-CRP levels, and increased peak expiratory flow (PEF). In a Survey Monkey questionnaire, 81% of participants who completed pre and post-testing expressed mild to strong positive opinions of the study contents, delivery, or impact, while 16% expressed mild negative opinions. Over the course of the year including the study, participant adoption of the MM practices showed a significant and moderately large correlation with overall health improvement; Pearson's *R* = 0.62, *p* < 0.005. These results support the hypothesized benefits of video-based MM training for this population. No adverse effects were reported.

**Clinical Trial Registration:** www.ClinicalTrials.gov, identifier: NCT02612389

## Introduction and background

### Morbidities in flight attendants exposed to second-hand cigarette smoke

Flight attendants (FA) who flew before the ban on smoking in commercial aircraft (implemented progressively from 1988 to 2000) present with many of the co-morbidities of chronic obstructive pulmonary disease (COPD) but their pulmonary dysfunction differs from the standard definition of this disorder. These FA, in good health at the time that they were hired, were exposed to second-hand cigarette smoke (SHCS) in the course of their often vigorous work activities, as well as to a wide range of other stressors [including interpersonal stress, aviation-associated threats and emergencies, disrupted diurnal rhythms and polluted air (1–4)]. Recent studies of this population (5–8) demonstrate significant rates of abnormal pulmonary function: air trapping, reduced flow at mid-volume, reduced exercise tolerance and significantly elevated rates of chronic bronchitis and sinusitis. They also exhibit increased rates of cardiac disease, depression and anxiety (9), sleep disturbances, skin and reproductive cancers and hearing loss (7, 10). Many of these symptoms are also co-morbidities of COPD (11, 12).

The magnitude and nature of these pulmonary abnormalities do not meet the standard criteria of COPD which is based on FEV1/FVC, the ratio of forced expiratory volume in 1 s (FEV1) to forced vital capacity (FVC) (12). The limitations of using the GOLD (12) standard defining COPD are increasingly recognized (13). Han et al. (14) have proposed that there is significant heterogeneity in the clinical symptoms of COPD and that a broader definition may be warranted to characterize its differing phenotypes. COPD is frequently characterized as being irreversible, yet these views generally predate current research that recognizes the heterogeneous nature of COPD (13) and the co-morbidity of post-traumatic stress disorder (PTSD) and COPD (or COPD-related symptoms) (15). Research into the effects of stress and trauma on pulmonary health, including insights into the embedding of fear responses by nicotine exposure, are changing the overarching paradigms about the nature of the lasting respiratory effects of tobacco smoke exposure (15–18).

### The role of cigarette smoke (CS) in disrupting autonomic nervous system (ANS) function

The role of FA is “to perform vital crewmember functions onboard air carrier aircraft, including emergency functions for aircraft evacuations, firefighting, first aid, and response to security threats” (19). Our work with this population of first responders has led us to examine the role of second hand cigarette smoke (SHCS) in autonomic nervous system (ANS) activation in this group. In addition to its role as a principal cause of COPD, cigarette smoke may contribute to mental health issues. Children (8–15 years old) exposed to secondhand smoke had higher rates of major depressive disorder and attention-deficit/hyperactivity disorder (20). Furthermore, there is a striking association between smoking and anxiety disorders, including PTSD (21). In emergency workers, smoking after exposure to a disaster was associated with increased PTSD symptoms; the authors suggest that smoking-related dysregulation of the hypothalamic-pituitary-adrenal axis contributes to increased PTSD symptoms (18). Studies in laboratory rodents show that nicotine is a causal agent of extended fear response. In mice, nicotine enhanced fear conditioning (22), delayed extinction of fear memories (23, 24), and disrupted safety learning (17, 25). While not directly tested, these effects of nicotine on fear learning and extinction could be related to altered hypothalamic-pituitary-adrenal function in those exposed to second hand smoke as well as active smokers. The resulting imbalance of the ANS may negatively impact the immune, cardiovascular, respiratory and musculoskeletal systems, and may reinforce the pathological changes triggered directly in the lungs by cigarette smoke (CS). This novel recognition of another mechanism for the negative effects of CS has not yet received the attention it deserves, and as far as we know, no therapeutic approaches specifically aimed at the autonomic effects of workplace exposure to SHCS have been proposed. Our selection of the meditative movement (MM) intervention and specific outcome measures are informed by the view that the autonomic effects of SHCS form a significant part of the lasting burden of exposure to SHCS.

### Qigong

Qigong, a traditional Chinese health practice, encompasses a large range of practices using specific postures, movements, breathing patterns and visualizations to address disease patterns and improve health. It has been used in China for hundreds of years for people with respiratory, autonomic and immune dysfunction (26). Many of these practices have a broad positive influence on a range of pathologies (27, 28), and tend to restore physiological functions to normal range (29). Qigong, as well as Tai Chi and Hatha Yoga, have been proposed to constitute a novel category of exercise, “meditative movement” (MM) (30). Specific MM practices have been shown to be an effective intervention for COPD (31–34) and to be equivalent or superior to conventional pulmonary rehabilitation in benefiting several of its symptoms (35, 36). MM has demonstrated benefits for the immune system, both reducing chronic systemic inflammation (37, 38) and increasing the effectiveness of acquired immune response to infection (39). MM practices help restore functionality to the ANS (40–43), and also may reduce depression and anxiety (38) and improve quality of life in many chronic diseases (44, 45). MM also induces positive states of mind through focusing awareness on interoceptive and proprioceptive experiences (46).

### Interoception

Interoception is the perception of the internal state of the body, and is recognized as important for emotional and physiological self-regulation (47, 48). Autonomic nerve pathways from the viscera link to the hypothalamus and to the insular cortex, facilitating physiological and affective homeostasis. Adequate interoceptive function is thus important for autonomic function as well as a sense of well-being. MM directly addresses interoceptive ability, and this may be one of the mechanisms for MM's beneficial effects on the ANS, immune system, and other physiological systems (48–50). We believe MM could prove to be of specific benefit to the particular problems experienced by FA exposed to SHCS, especially in view of the possible linking factor of autonomic imbalance.

### Aim

Our aim was to determine the effectiveness of a digitally delivered MM training, as compared to a control group that received health education videos, by pre-intervention and post-intervention testing of pulmonary, autonomic, interoceptive, and immune function in a cohort of FA who were previously exposed to occupational SHCS. We sought to determine whether this specifically adapted MM training can be effectively delivered digitally, providing the benefits of MM without face-to-face instruction.

## Methods

The present paper details Phase II of a 2-Phase study. This followed Phase I, a single-arm non-randomized pilot study in which we developed and tested methods for MM intervention using face-to-face classroom instruction. The full protocol and results from Phase I have been published (51). Phase II is a randomized controlled trial comparing MM delivered by video only, to a health education (HE) intervention. This study was approved by Dartmouth College IRB (CPHS #28572). Both phases of the study are registered at Clinical Trials.gov under the code: https://clinicaltrials.gov/ct2/show/NCT02612389/. As approved by the Dartmouth CPHS, all participants gave written consent using IRB-approved informed consent forms (52) when they enrolled in the study. Participants were assigned alpha-numeric code numbers to provide anonymity and protect the privacy of study volunteers.

### Participants

#### Recruitment

Fifty-one FA were recruited from throughout the continental US, in particular the Northeast and areas around Atlanta GA, Miami FL, San Francisco CA, Seattle WA, and Portland, OR. Primary outreach was through the networks of FA that we had already established in Phase I, as well as through existing flight attendant organizations, social media, flight attendant publications. Our collaborators at Harvard School of Public Health also provided support. Social media support was provided by the Department of Health Behavior at the Roswell Park Cancer Institute in Buffalo, NY.

#### Inclusion and exclusion criteria

All participants were required to be non-smoking former or current flight attendants, who had been exposed to SHCS for at least 5 years while flying. In addition, participants were required to have devices for accessing audio and video content and instruction, and to be willing to use them. Pregnancy or planned pregnancy, as well as cognitive impairment, severe emotional problems, or physical inability to perform the exercises, were grounds for exclusion. Participants were asked not to modify their lifestyle significantly during the study period apart from the practice required by the study. Since each participant acted as his/her own control, we did not exclude participation on the basis of medication use.

#### Randomization and controls

In this randomized controlled trial (RCT), participants were stratified by region and randomized to the intervention group or to the control group, using the covariate adaptive randomization method (CARM) (53). CARM was used because it accommodates recruiting and testing over an extended timeframe. Volunteers assigned to the MM interventional group received weekly assignment with specific MM training videos. The control group received health education (HE) videos. Blinding participants to the intervention was not practicable. Tests were scored by assistants who were blinded to the assignment of study participants.

### Intervention

#### Experimental design

**Figure 1:**
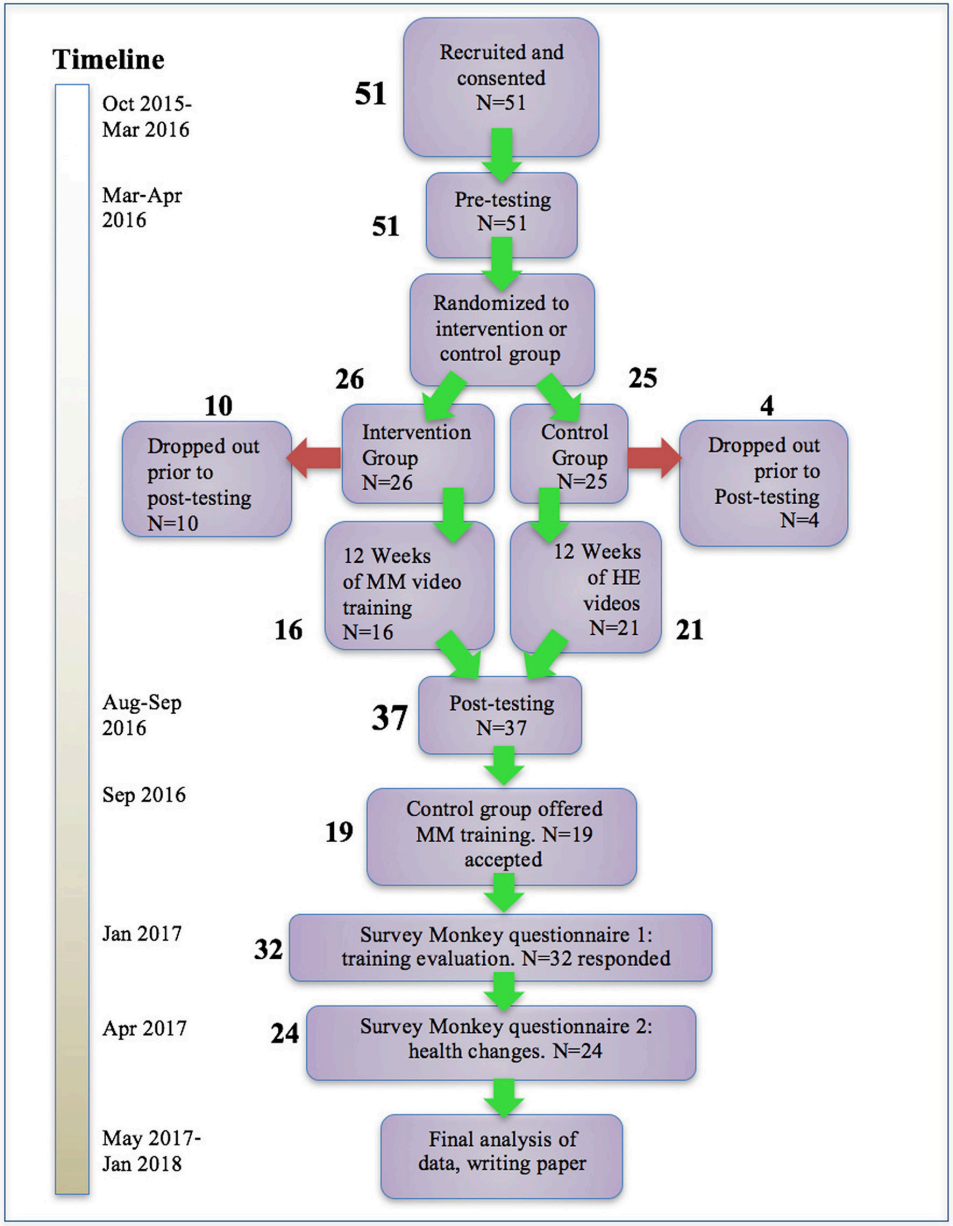
Participant flow and timeline. After recruiting and pre-testing, participants were randomized to intervention and control groups. Following the training period, participants were tested again, and the control group participants were offered the opportunity to receive the same MM training. After all participants had completed the training, two final questionnaires were administered. Dropouts and numbers responding to questionnaires are shown.

In Figure 1, experimental design for this study is shown. Following recruitment, enrolling, and consenting, pre-intervention testing was administered at locations throughout the country convenient to participants. Over a 2-h period, participants completed the questionnaires, cardiovascular measures were taken, and various tests were administered as described below.

Participants were then randomized to a MM intervention group or a control group receiving HE videos, which were selected Ted Talks available through YouTube. Over ~4 months, participants were assigned ~10 h of video instruction in MM exercises. Exercise selection was based on experience from Phase I. The video assignments were sent out each week via email with instructions to watch and review selected videos online. The control group also received an email each week assigning a HE video. HE videos dealt with general health questions, but did not contain instructions on any form of practice. Both groups were also completed weekly Survey Monkey questionnaires about their weekly participation. After the training period, the same tests were administered, and the data was analyzed.

At the conclusion, the participants in the control group were offered the opportunity to engage the MM training by viewing the MM videos, with the same weekly structure as was used for the intervention group. After this group had completed their training, all those who received the MM videos, including intervention and control group participants, were asked to complete a Survey Monkey questionnaire evaluating the training; and 6 months after the conclusion of training, all participants were asked to complete a second survey about changes in physical and affective health over the previous year.

#### Implementation of intervention

**Figure 2:**
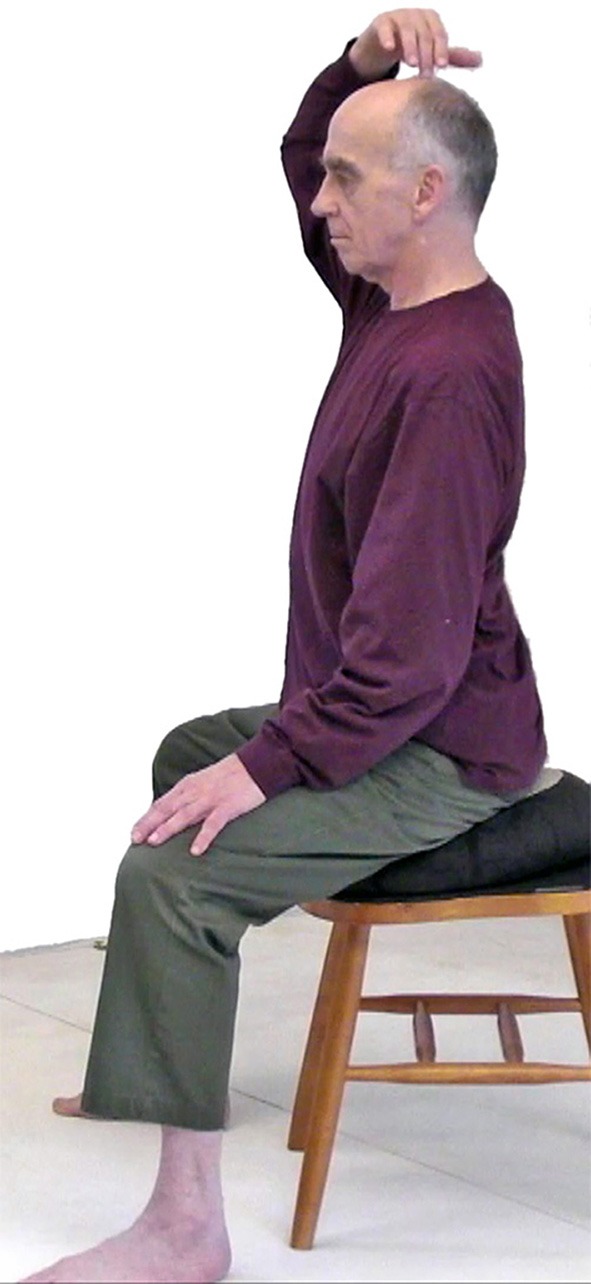
A typical MM practice. One of the authors demonstrating an MM exercise, showing how to sense correct alignment with gravity while seated.

The MM instructional videos developed for the Phase II MM intervention were based on our work in Phase I (51), in which we determined the most appropriate practices for this population of FA. These included simple postural practices, such as standing and sitting, moving practices, such as walking, and simple breathing practices. The practices involved static postures or slow and gentle movement. Participants were asked to direct their attention to environmental, interoceptive and kinesthetic awareness, and to focus on specific mental images. One advantage of this form of practice is that it can easily be integrated into daily life, thus making minimal time demands, and participants were instructed to implement this integration. Figure 2 shows one of the authors (PP) demonstrating a practice of awareness of balance while sitting. Access to the videos is available (54–56).

The videos used for the control group were Ted Talks available on YouTube, pertaining to general health and not including the teaching or encouragement of specific health practices. Each week all participants received an email asking them to view specific videos; the MM videos were distributed to participants either via links to Vimeo files or by mailed DVDs, depending on the preference of participants; and the control group participants were provided with links to health education Ted talks on YouTube.

Each week study participants were sent Survey Monkey questionnaires with follow-up emails or phone calls if participants did not respond. In the questionnaires they were asked to report on levels of participation and provide feedback on their experiences while doing the MM practices. Feedback was regularly reviewed to evaluate the use, compliance and acceptability of each of the practices.

Harmful side effects are very rare in the practice of MM (57), but participants were instructed to immediately discontinue the exercises in case they should experience dizziness, rapid or irregular heart-beat, chest pain, pressure in the head or headache, significant pain anywhere in the body or sudden or excessive dyspnea.

### Outcome measures

#### Primary outcome measures

The guidelines of the American Thoracic Society (ATS) were followed when conducting the six-minute walk test (6MWT). These tests were conducted on a level indoor surface, free of obstructions. Trained research technicians were present to assist with any difficulties experienced by participants. The distance walked in 6 min by each participant was measured and recorded (58–61).

Blood samples were obtained to measure High Sensitivity C-reactive protein (hs-CRP), a useful marker for systemic inflammation (62). This was obtained by finger-stick blood draw method, collected onto a prepared blood card. After allowing the blood spots to dry overnight the filter cards were stored frozen until they were shipped to ZRT Laboratory where they were analyzed by immunoassay as previously described (63).

#### Secondary outcome measures

Blood pressure was recorded in a seated position from the dominant arm, after the participant had been at the testing center for over 30 min. At least two readings were taken, 2 min apart, and if the readings differed by more than 10 points, a third reading was taken and the average value obtained and recorded.

The COPD Assessment Test (CATest) (64, 65), a short questionnaire used to evaluate the perceived impact of respiratory dysfunction, was utilized.

Spirometry was performed according to the ATS guidelines (66) using specific validated spirometric cut points (67). Standard measures recorded were FEV1 (forced expiratory volume in 1 s), FVC (forced vital capacity), forced expiratory flow between 25 and 75% of capacity (FEF 25–75), peak expiratory flow (PEF) and flow/volume curves, using the EasyOne Plus Frontline spirometry system (ndd Medical Technologies, Andover, MA).

To detect symptoms of autonomic dysfunction, subjects completed the self-report COMPASS 31 (68).

The Zung Self-Rating Depression and Anxiety Scales (self-report instruments) were employed (69, 70). Subjects completed these questionnaires in the presence of a research team member.

Subjects completed the Multidimensional Assessment of Interoceptive Awareness (MAIA) (71). The degree of awareness of interoceptive cues, and the level of comfort with these interoceptive cues are evaluated by the MAIA. The ten different dimensions measured include: Noticing, Not Distracting, Not Worrying, Attention Regulation, Emotional Awareness, Self Regulation, Body Listening and Trusting.

Blood samples were obtained using finger-stick procedure, and analyzed at ZRT Labs, Beaverton, Oregon, as described in Payne et al. (51). Diurnal urine and saliva samples were also collected. Subjects were given collection kits and instructed in their use, and mailed the samples to the researchers. Samples were stored at −80°C and sent in batches to ZRT Lab for analysis by mass spectrometry. For a full listing of biomarkers tested, see our earlier protocol paper (51).

##### FA survey monkey questionnaires

Participants in the MM study were surveyed weekly to determine the level of participation and follow through with assigned videos. Non-compliant subjects were reminded first by email and then by phone calls. Participants in the control group were also surveyed and followed on a weekly basis.

In addition to the weekly progress reports, participants were asked to complete two questionnaires. Survey Monkey questionnaire 1: participants who had received the MM training videos (including both those in the initial MM intervention and those in the control group who chose to receive the videos after completion of Phase II) were asked the following five questions about their response to the videos, via a Survey Monkey questionnaire. All respondents were included in the subsequent data analysis.

1: Please give general comments on the practice.
2: Please critique the video presentation.
3: Are you willing to continue participating in studies of MM?
4: Are you finding that you are more aware of your body after participating in the training?
5: Do you feel more able to cope with stress after this training?

Each response was scored by a neutral blinded third party as very positive, positive, neutral, negative, and very negative, using numbers from 2 to −2, and the scores summed for each participant.

The second Survey Monkey questionnaire was administered 6 months after completion of the training. All participants who had received the training videos were asked via Survey Monkey about their continuing practice of the various exercises, rating each of 14 exercises as “not doing it,” “doing occasionally,” or “doing 3 times a week or more.” In addition, participants were asked about their health changes over the prior year. Each of 11 physical health symptoms (sleep quality, balance, endurance, breathlessness, joint pain, digestion, respiratory infections, dry mouth, cardiovascular disorders, blood pressure, overall health) and four affect-related items (mood, security, happiness, dealing with stress) were rated as “never a problem,” “worse compared to a year ago,” “about the same as a year ago,” or “improved compared to a year ago.”

### Statistical methods and scoring

We determined the required sample size for statistical significance based on a predicted difference of 46 meters in the 6MWT and an assessment of previous similar studies. Using this criterion, 20 participants in each group were needed to achieve a statistical power of 80% at a significance level of 5%. To compensate for anticipated drop-out, we recruited a total of 51 participants. Descriptive statistics, including mean, percent change, correlation coefficients, and *p*-values, were used to describe and summarize data. In cases where data for a subject on one outcome measure is missing, we have chosen to eliminate that subject from the calculations for that measure. Significance levels for changes in measures from pre- to post-intervention test of the MM group as compared with the control group were determined using ANOVA. *P* < 0.05 was selected as the threshold for significance. Raw data and statistical calculations made are available.

The results from the Survey Monkey questionnaires were treated as categorical data. In Survey Monkey questionnaire 1, each response was scored by a neutral third party as very positive, positive, neutral, negative, and very negative using numbers from 2 to −2, and the scores summed. In the question about willingness to continue, a “yes” was scored as +2, a “no” as −2, and a “don't know” as zero. Possible total scores for the five questions ranged from 10 to −10. These results are presented in a bar graph without the use of *p*-values as these were not deemed necessary.

In Survey Monkey questionnaire 2, for the health portion, responses of “never a problem” and “about the same as a year ago” were assigned a value of zero; “worse compared to a year ago” was scored as negative 2; and “improved compared to a year ago” was scored as positive 2. Scores for each person were summed in an affective health change score, a physical health change score, and a total health change score. In the exercise adoption portion, we wished the total score to reflect the amount of time each participant spent per week on practice. Therefore, we scored “not doing it” as zero, “doing occasionally” as 1, and “doing 3 times a week or more” as ten. Exercise adoption scores were summed for each person, and Pearson's R correlation coefficients, and *p*-values were determined.

## Results

### Participants

#### Participant flow

Figure 1 above shows numbers of participants recruited and consented, number of drop-outs, and numbers completing the study. Fifty-one flight attendants met the inclusion criteria and were recruited and consented. They were then stratified by geographic area and randomized to an MM intervention group (*N* = 25) and a health education control group (*N* = 26). The two groups began receiving the appropriate videos as well as regular Survey Monkey questionnaires. Prior to the completion of the post-intervention testing, 10 participants in the MM group and 4 in the control group discontinued the study due to stated lifestyle conflicts. Sixteen completed the MM portion and 21 completed the control portion for a total of 37. When offered the opportunity to receive the MM training after the completion of post-testing, 19 participants from the control group opted to do so, and over the following 4 months received the same sequence of MM training videos as was used in the main intervention. Two final Survey Monkey questionnaires were sent to all those who had received the training. Results were then analyzed.

#### Baseline clinical characteristics and demographics

The characteristics of the 51 consented and pre-tested recruits are shown in Table 1. While every effort was made to reach out to a diverse population, all of those consented for this study were female and White. This lack of diversity is consistent with the findings of McNeely (72) who reported that only 11.5% of former or current flight attendants between the ages of 50 and 90 are non-White. McNeely also reported that 87% of US flight attendants between 50 and 90 are female (72). This helps to explain the low level of diversity within the study population.

**Table 1:** Baseline characteristics of subjects who completed the pre-testing phase of the study.

	**MM Intervention**	**Control group**	**Dropouts or did not complete post-testing**
Total randomized (completed pre-testing)	26	25	n.a.
Numbers in each group	16	21	14
Female	100%	100%	100%
Average age	68	68	67
Age range	62–74	55–79	51–79
Body mass index average	24.3	24.6	25
BMI range	18.5–33.7	20–29.7	17.1–30.7
White	16	21	14
African-American	0	0	0
Hispanic	0	0	0

*51 subjects were consented and tested at the beginning of the study, and of these, 37 completed the study*.

Baseline clinical characteristics of the two groups differed substantially. Due to random variation, the control group was healthier than the intervention group on almost all health-related measures. The control group scored more than 5% lower on the CATest, Zung Anxiety, Zung Depression, COMPASS 31, hs-CRP (all indications of better health); and had higher scores on the MAIA (especially body listening) and higher blood levels of testosterone and DHEAS. The only measure possibly indicative of lower health levels in the control group were lower melatonin levels. This data appears in Table 2.

**Table 2:** Baseline clinical characteristics.

**Measure**	**MM**	**Control**	**Control minus MM**	**Control minus MM %**
COPD-short	8.88	7.24	−1.64	−18%
FEV1	90.25	89.38	−0.87	−1%
FVC	89.44	89.95	0.51	1%
FEV1/FVC	100.50	99.05	−1.45	−1%
FEF 25–75	91.88	90.24	−1.64	−2%
PEF	95.69	97.76	2.07	2%
Zung anxiety	32.94	31.20	−1.74	−5%
Zung depression	36.63	34.70	−1.93	−5%
COMPASS31	16.81	14.05	−2.76	−16%
MAIA: overall	21.22	26.75	5.53	26%
Noticing	2.77	3.45	0.68	25%
Not distracting	2.20	2.45	0.25	11%
Not worrying	2.87	3.00	0.13	5%
Attention regulation	2.64	3.25	0.62	23%
Emotional awareness	3.47	3.98	0.51	15%
Self regulation	2.97	3.53	0.56	19%
Body listening	2.20	3.17	0.97	44%
Trusting	3.49	4.10	0.61	18%
6MWT-meters	538.90	546.90	8.00	1%
Blood pressure: diastolic	76.56	79.76	3.20	4%
Systolic	123.31	124.33	1.02	1%
Resting heart rate	67.31	67.90	0.59	1%
hs-CRP	1.63	1.52	−0.11	−7%
Testosterone	16.93	21.10	4.17	25%
DHEAS	2.13	3.68	1.55	73%
1st AM urine cortisol	29.85	43.05	13.21	44%
1st AM urine melatonin	25.31	22.05	−3.26	−13%

*The right column shows the percentage difference between the intervention and control groups average value at baseline. Green boxes indicate measures possibly indicative of better health; red boxes indicate potential indicators of worse health*.

#### Adverse events

Reports of adverse side effects from MM are rare (57). Consistent with this, no adverse events were reported by any participant enrolled in this study.

### Outcome measures

#### Detailed results of pre-post testing

Results are shown in Table 3. Analysis of variance (ANOVA) revealed statistically significant improvements in the MM group compared to the control group, of the 6MWT, the MAIA score, and the CATest, indicating improved endurance, interoceptive awareness, and pulmonary function. All the MAIA sub-scores improved substantially (+10% or above) compared to the control group, with the exception of Noticing and Emotional Awareness; there were no substantial increases in MAIA sub-scores in the control group. However, only the 26% increase in the MM group of Self-Regulation reached significance (*p* < 0.05). A large increase in DHEAS levels in the MM group as compared to the control group almost reached stated significance (*p* = 0.0566). Among the changes not reaching statistical significance were: a substantial (29%) reduction in hs-CRP in the MM group compared to a slight (6%) decrease in the control group; the COMPASS 31, a measure of autonomic dysregulation, increased by 7% in the control group (indicating increased autonomic dysregulation), and decreased by 9% in the MM group; and the Zung Anxiety score fell by 10% in the MM group and by 4% in the control group. There was also a non-significant slightly reduced resting heart rate in the MM group as compared to the control group, and slightly increased peak expiratory flow (PEF) in the MM group coupled with slightly decreased PEF in the control group. There was a large increase in blood testosterone levels in both groups.

**Table 3:** Outcome measures.

**Outcome measure**		**% Change in MM group**	***p*-value (ANOVA)**	**% Change in HE group**
Primary	6MWT	+7	*p* < 0.005^**^	−2
	hs-CRP	−29	*p* = 0.76 ns	−6
Cardiovascular	Avg systolic BP	0	ns	2
	Avg diastolic BP	2	ns	−3
	Resting HR	−4	*p* = 0.6 ns	0
	% Change HR after 6MWT	18	*p* = 0.1	−18
Pulmonary	CATest score	−32	< 0.01^*^	+12
	FEV1	0	ns	−1
	FVC	0	ns	−1
	FEF 25–75	−1	ns	−3
	PEF	+2	ns	−2
Autonomic	Compass 31	−9	*p* = 0.1117	+7
Humoral	Vit D level	−2	ns	4
	Testosterone	+44	ns	+41
	DHEAS	42	*p* = 0.0566^◇^	−10
Interoceptive	MAIA	24	*p* < 0.005^**^	−1
	Noticing	9	ns	6
	Not distracting	13	*p* ~ 0.1	−16
	Not worrying	11	*p* ~ 0.1	−4
	Attention regulation	14	ns	4
	Emotional awareness	7	ns	2
	Self regulation	26	*p* < 0.05^*^	2
	Body listening	28	*p* ~ 0.1	−7
	Trusting	21	*p* ~ 0.1	6
Affective	Zung anxiety	−10	*p* = 0.234 ns	−4
	Zung depression	−8	ns	−8

*Percent change in values for the intervention group and for the control group are shown as well as p-values determined by ANOVA. Full pre- and post-test values are available as Supplementary Materials*.

** , ≤ 0.01 significance level;

* , ≤ 0.05 significance level; +, trending;

◇ *, almost significant; ns, not significant*.

#### Survey monkey questionnaire 1

After all training was completed (Jan 2017), the 35 participants who had received the MM video training (including 16 in the initial MM intervention and 19 in the control group who chose to receive the videos after completion of post-testing) were asked the following five questions about their response to the videos, via a Survey Monkey questionnaire. Thirty-two participants responded to the questionnaire.

1: Please give general comments on the practice.
2: Please critique the video presentation.
3: Are you willing to continue participating in studies of MM?
4: Are you more aware of your body after participating in the training?
5: Do you feel more able to cope with stress after this training?

As described under section Statistical Methods and Scoring, above, each response was scored by a neutral blinded third party.

**Figure 3:**
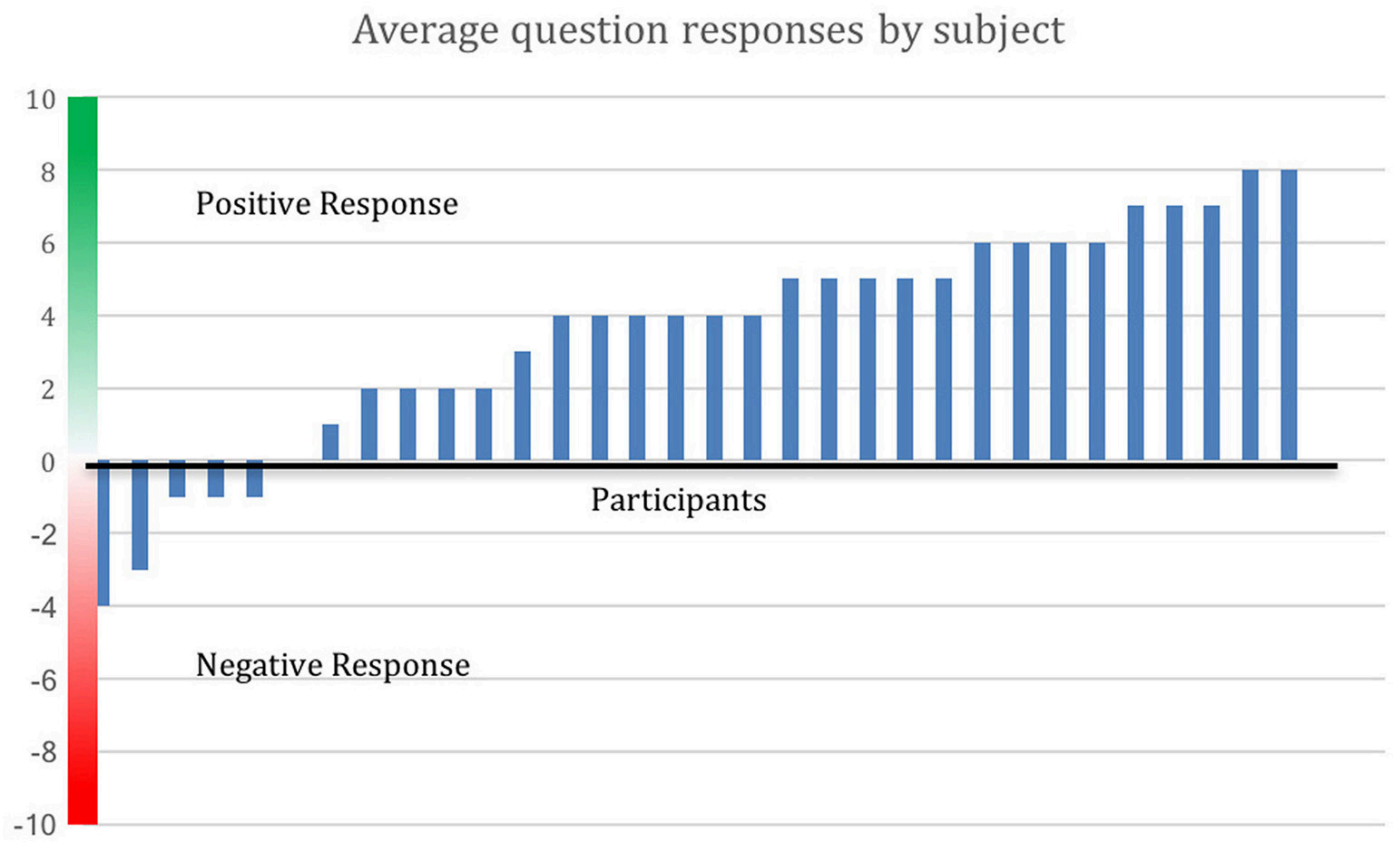
Positive evaluation of training by 81% of participants. Average response to the Survey Monkey questions is shown by participant. Out of 32 respondents (16 from the MM group and 16 from the control group who opted to receive the MM training), 26 made an average positive evaluation. Distance above the *x*-axis indicates degree of positive response, below the *x*-axis indicates negative response.

**Figure 4:**
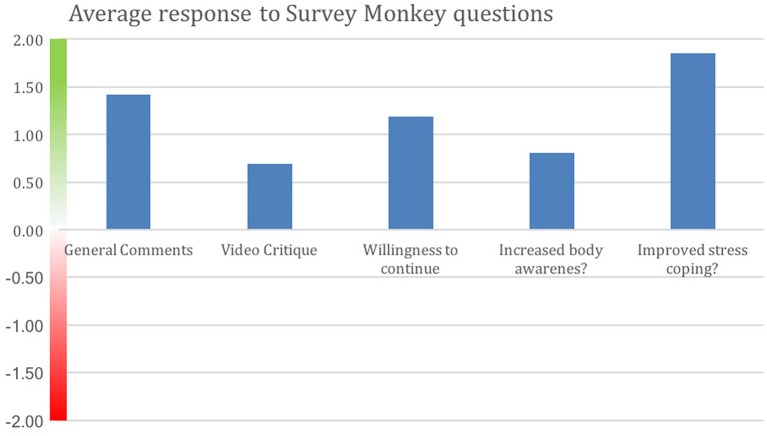
Positive average response by all participants to each Survey Monkey question. Improved stress coping received the strongest positive response. Y-axis indicates degree of negative or positive responses. 16 of the MM intervention participants and 16 from the control group who opted to do the MM training responded. Not all participants answered all questions.

Figure 3 shows degree of positive rating from each participant; 81% of participants rated the overall experience positively. Average scores for each question are shown in Figure 4. Scores for each of the five questions could range from 2 to −2. Not all participants responded to all questions. We note that numerous participants remarked on increased stress resilience and positive body awareness, which appears to be reflected in the increased MAIA sub-scores of Self-Regulation (+26%) and Body Listening (+26%).

#### Survey monkey questionnaire 2

Six months (Sept 2017) after the completion of training, participants were again surveyed to determine if there were perceived changes in health or wellbeing. As described above in section Statistical Methods and Scoring, the “Health: Physical” and “Health: Affective” portions were scored separately and also combined for a total health change score. Scores for each person reflected the change of their level of health since 1 year ago, and were therefore roughly reflective of health change over the duration of the study. Exercise scores were indicative of the degree to which the subject adopted the MM practice and continued beyond the end of the study. All of those answering had received all the MM video materials and instructions at some time during the study. We excluded those who experienced a major health crisis, such as major surgery or severe illness, over that period of time, on the grounds that these events induce disturbance that is not relevant to, and obscures, the factors of the study. Twenty-four respondents are included in the analysis, 12 from the intervention group and 12 from the control group who opted to do the MM training.

**Figure 5:**
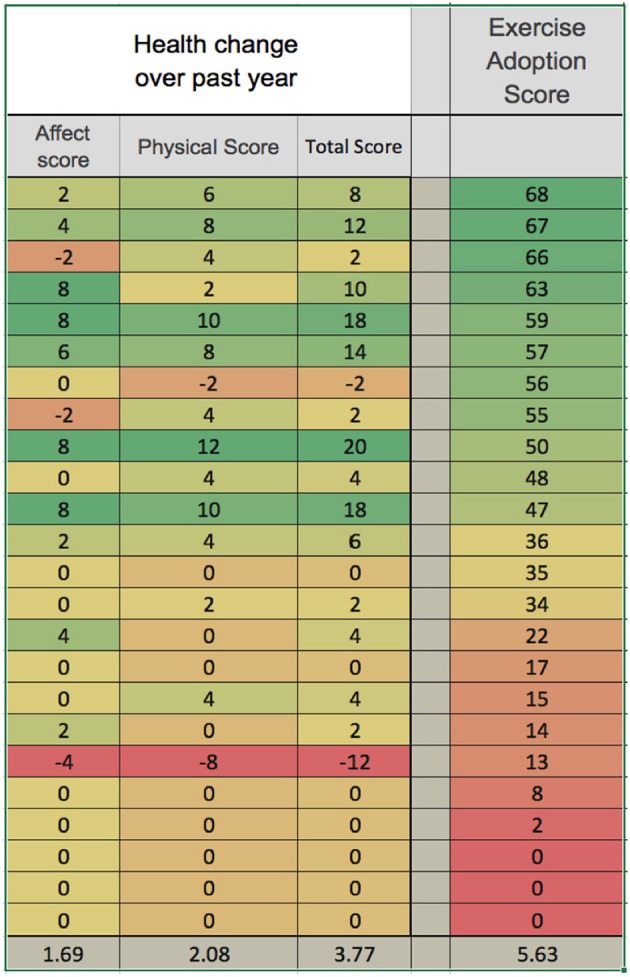
Reported adoption of MM exercises is correlated with health improvement over the prior year. Reported health change over the duration of the study is shown in graphical form, with green the most positive and red the most negative. Physical health change and affective health change are shown separately and summed for total health score. Exercise adoption score is also shown, and the data are ordered according to this score. Averages are shown at the bottom. Table 4 below shows correlational analysis.

Figure 5 shows the results in graphical form, and Table 4 shows correlation and significance. Pearson's correlation analysis shows moderate and significant positive correlations, indicating that the amount of variance in year-to-year health change attributable to interaction between the variables is approximately one-third. The correlation to the physical health score is distinctly greater than to the affective score.

**Table 4:** Exercise adoption is positively correlated with health improvement.

	**Pearson's *R***	***R* squared**	***P*-value**	**Significance level**
Exercise adoption to total health change	0.5822	0.34	0.003	*P* < 0.005
Exercise adoption to physical health change	0.6163	0.38	0.001	*P* < 0.005
Exercise adoption to affective health change	0.4445	0.20	0.03	*P* < 0.05
Physical health change to affective health change	0.7257	0.53	0.000	*P* < 0.001

*Statistical analysis of the results of SM questionnaire 2 are shown. Pearson's R indicates the strength of the correlation; R-squared indicates the percent of variance accounted for by the interaction of the variables, significance level is also given. Highly significant moderate positive correlations are shown between reported degree of adoption of the MM exercises, and reported changes in physical and affective health over the prior year including participation in the study*.

#### Graphical summaries of key results

**Figure 6:**
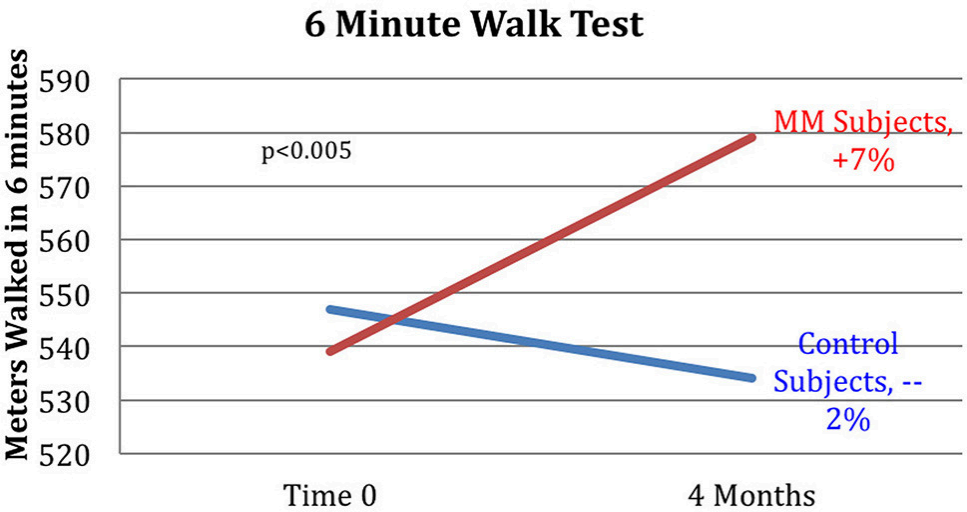
Improved 6MWT score for MM group vs. control group. Subjects in the MM group averaged a 7% increase in distance covered in the 6MWT over the 4 months between pre- and post-testing. Control HE subjects averaged a −2% change during the same time frame. ANOVA yields a *p* < 0.005.

**Figure 7:**
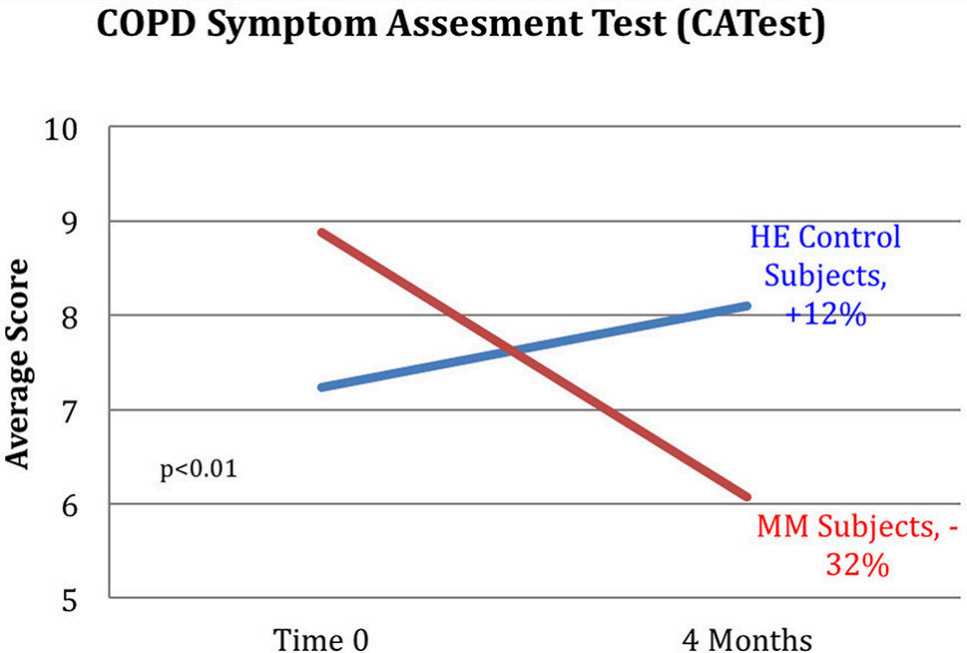
Reduction in CATest scores for MM subjects. MM subjects scored a 32% decrease on the COPD assessment test indicating decrease in symptoms between pre and post-testing while those in the control HE group averaged an increase of 12% in COPD assessment test symptom score. ANOVA yields a *p* < 0.01.

**Figure 8:**
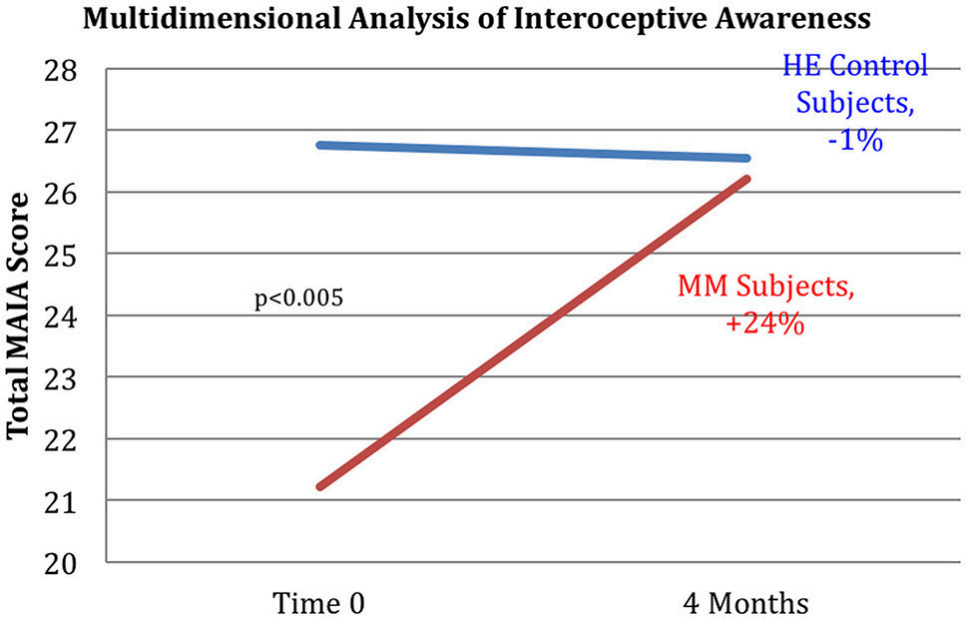
Improved interoception in MM group as shown by MAIA score. Subjects in the MM group averaged a 24% increase in MAIA overall interoceptive awareness score over the 4 months between pre and post-testing. Control subjects averaged a 1% decrease during the same time frame. ANOVA analysis yields a *p* < 0.005.

**Figure 9:**
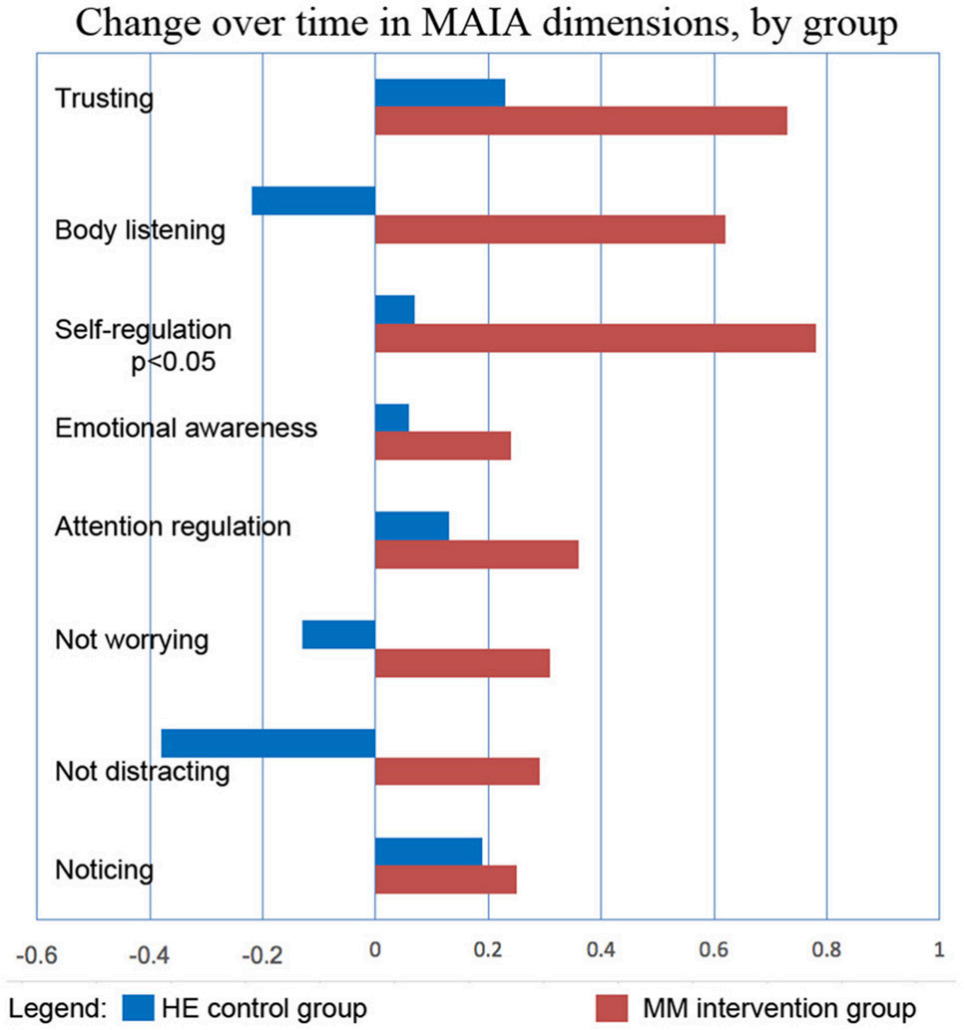
MM intervention group shows substantially more improvement in all sub-scores of the MAIA, as compared with the HE control group. The changes in MAIA sub-scores over the 4 months period are shown next to each other for the two groups. The difference between sub-scores for Self-regulation reached significance at *p* < 0.05 (ANOVA).

**Figure 10:**
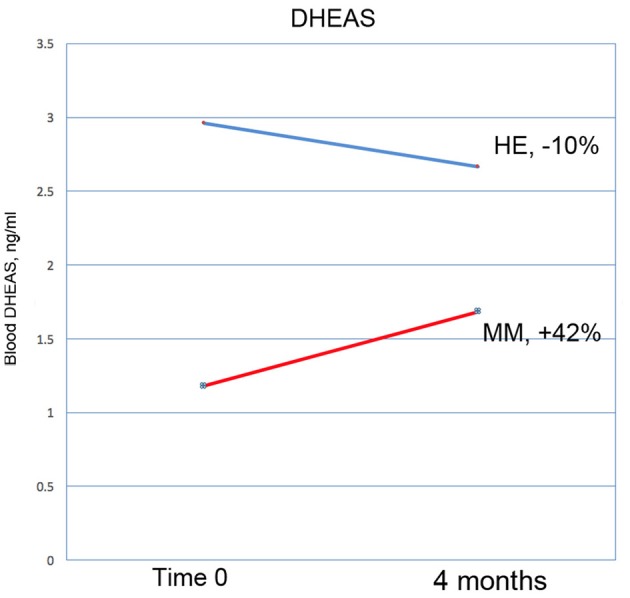
DHEAS increased by 42% in the MM intervention group over the 3 months of the study, compared with a drop of 10% in the HE control group. This result nearly reaches significance at *p* = 0.0566 (ANOVA).

**Figure 11:**
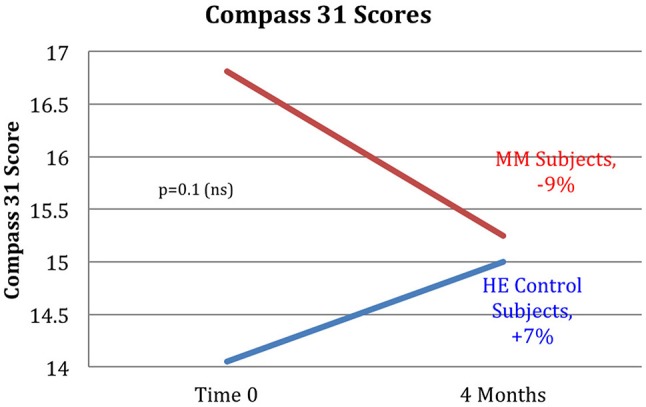
The MM intervention group showed improved autonomic function over the study period, the HE control group showed reduced function. The COMPASS 31 questionnaire measuring autonomic function shows a substantial difference between the groups. The MM group improved by 9%, while the control group worsened by 7% over the study period. These results did not reach stated significance threshold (*p* = 0.1).

Key results of the study are summarized in graphical form in Figures 6–11. These figures reflect changes in test results measured before and after the MM intervention.

## Discussion

### Summary

Our results demonstrate improved exercise tolerance and endurance, reduced COPD-related symptoms, and improved interoception, especially in the ability to handle stress. This supports our hypothesis that video-delivered MM training can produce similar health benefits to those found in Phase I with in-person instruction. Phase I results had shown clinically relevant and statistically significant improvements in the 6MWT, hs-CRP, systolic BP, resting HR, COMPASS31, MAIA, and Zung Anxiety Inventory, as well as clinically significant improvement in the CATest. In Phase II, the COMPASS 31 (a measure of autonomic function), hs-CRP (a biomarker of systemic inflammation), resting heart rate, and anxiety, all showed substantial improvement in the same direction as found in the Phase 1 study, but without reaching statistical significance. The changes in symptoms of dyspnea, as well as improved endurance demonstrated by the 6MWT results, support our hypothesis that MM practice can improve pulmonary health. These results are in line with other studies of the effects of MM, specifically on respiratory disease (33) as well as on other health issues (37). Reduced exercise endurance (as measured by the 6MWT) is a strong risk factor and co-morbidity for a variety of pathologies, especially in the elderly, suggesting that MM practice may have preventive and ameliorative health effects in FA exposed to SHCS and possibly in other populations.

### Outcomes

Pre and post-intervention testing showed that the MM intervention was effective and that it significantly changed results of the 6MWT, one of the primary outcomes. The MM intervention training contained no form of resistance training or aerobic exercise and the underlying physiological mechanisms of these changes remain uncertain. We have speculated that they are due to changes in the autonomic nervous system. In the current study, autonomic function, as measured by COMPASS 31, showed improvement in the MM interventional group, while scores in the control group indicted poorer autonomic function (as was reported in Phase I) although this did not reach statistical significance. We note that in both this study and in Phase 1 there was a small increase in peak expiratory flow (PEF); this did not approach significance, but we speculate that spirometrically detectable changes may require longer or more intense practice. The MM intervention training contained no form of resistance training or aerobic exercise and the underlying physiological mechanisms of these changes remain uncertain. We have speculated that they are due to changes in the autonomic nervous system. In the current study, autonomic function, as measured by COMPASS 31, showed improvement in the MM interventional group, while scores in the control group indicted poorer autonomic function (as was reported in Phase I) although this did not reach statistical significance.

The MAIA measures the degree of awareness that an individual has of their body feelings. This self-report instrument has ten sub-scores that test various aspects of interoceptive awareness. Tests of subjects in the MM training group indicated a substantial and significant increase in MAIA score. A capacity for self-regulation is hypothesized to relate to interoceptive awareness (48, 73). This capacity includes the ability of the anterior cingulate gyrus and the prefrontal cortex to modulate arousal in the hypothalamus and limbic system, central components of the ANS (48, 73, 74). The hypothesis that the MM intervention acted in part by improving autonomic functioning via increased top-down control, is supported by the significant 24% increase in the MAIA score. The ability of the autonomic nervous system to receive afferent information about the physiological state of the body, primarily via the vagus nerve, is known to be crucial to autonomic regulation of organ function, which plays a significant role in COPD (75). Conscious interoception, the ability to be consciously aware of the body sensations which bring information about the visceral state of the body, has been shown to play a vital role in self-regulation and emotional self-control (47, 49, 50, 76). This strongly impacts the ability to take voluntary, conscious self-regulatory actions to cope with stress in a more resilient way. We note that, in this study, not only did the overall MAIA score improve significantly, but also the sub-score pertaining to self-regulation carried the majority of the improvement (Although all the sub-scores improved in comparison to the control group, only the self-regulation score reached statistical significance). These results suggest that improved interoceptive capacity may be part of the mechanism whereby MM improves the functioning of the ANS.

Kinesthetic interoception also plays a prominent role in the body's maintenance of mechanically efficient posture and movement, which are strongly emphasized in the MM training. Improved body mechanics and postural balance is also a plausible explanation for the dramatic improvement in 6MWT performance, which we have observed in both Phase I (38) and II of this study.

Several of the secondary outcome measures changed in the same direction as in the Phase I study, but without reaching statistical significance. In each case the change in the intervention group was in the direction of improved health, and in the control group in the direction of worse health or minimal change. These include hs-CRP, COMPASS 31, Zung Anxiety, and resting heart rate. In this cohort of women, average age 68, the expectation is of gradual decline in health. Despite being in better health to begin with, the control group demonstrated the expected decline in almost all measures: a 2% reduction in the 6MWT, no change in resting heart rate, a 12% adverse change in the CATest score, a 7% increase in measure of autonomic dysfunction by COMPASS 31, a slight reduction in PEF, and −1% change in the MAIA. Both groups had reduced hs-CRP levels, but the control group had substantially less reduction than the MM group (−6 vs. −29%). Similarly, both had reduced anxiety, but the MM group showed over twice as much reduction as the control group (−4 vs. −10%).

Despite the lack of statistical significance of some of these results, these outcomes suggest a pattern in the intervention group of generally increasing health, where one would expect an age-related decline, as seen in the control group. These results call for further research with further refinement of instructional methods and a larger cohort.

Interestingly, blood levels of DHEAS increased substantially in the MM group as compared with the control group; a 42% increase vs. a 10% decrease, at a significance level of *p* = 0.057 which closely approached defined significance. In the Phase I study, DHEAS increased by 37%, but the result did not reach significance. DHEA is an important precursor of a number of steroid hormones in the body. It declines significantly with age, and low levels are associated with an increase in neurodegenerative conditions, possibly due to its affinity with neurotrophin receptors (77). There is evidence that it increases brain levels of neuro-protective substances, such as nerve growth factor and brain-derived neurotrophic factor (78). In a rat model of Alzheimer's disease it demonstrates neuro-protective function (79), and may be involved in the mechanisms of depression (80). Although there is evidence that DHEA supplementation has no significant benefits in healthy people (81), it can be of benefit in certain disorders [for example (82, 83)]. Meditation (84) and exercise have been shown to increase DHEA levels; for a review, see (85). We suggest that in future similar studies DHEAS should be considered as a useful objective marker of improved health in this kind of intervention.

### Survey monkey questionnaires

Survey Monkey questionnaire 1 demonstrated that the participants had a substantially positive view of the study, with some criticisms of the videos for being too long and not sufficiently engaging. Their descriptions of their experiences with the practices were highly positive; in view of the MAIA results, it is interesting that participants strongly affirmed their perception of an increased ability to handle stress (self-regulation) and almost universally expressed a desire to continue with a similar study.

Survey Monkey questionnaire 2 showed a significant (*p* < 0.005) and moderate correlation (Pearson's *R* = 0.58) between the degree of adoption of the MM exercises, and reported health changes over the prior year, which included the duration of the study. Given the number of factors which can influence health changes over the course of a year, we regard this correlation as an important result supporting the overall positive impact of the MM training. We eliminated from the analysis participants who experienced a major health event over the year, such as hip or knee replacement surgery, on the basis that such an event would not be influenced by the study and would introduce irrelevant confounding factors. It seems unlikely that this would unduly distort our results. We do however recognize that causal effects can go both ways: participants in better health might have been more likely to adhere to the MM practices. Therefore, we regard this result as supportive but not definitive.

### Nicotine and the autonomic nervous system

The harmful effects of cigarette smoke on the lungs and cardiovascular system are well-known. The short-term effects of CS and nicotine on the ANS are less widely known but well-studied: inhaled cigarette smoke causes immediate effects on the autonomic nervous system, including increased heart rate due to catecholamine secretion, temporary mood elevation probably due to dopamine and DHEA, and stimulation of the hypothalamic-pituitary-adrenal (HPA) axis, which triggers the release of the “stress hormone” cortisol (86). Chronic smokers have elevated levels of cortisol and disturbances in the diurnal cortisol rhythm (87). It is known that chronic stress impairs recovery from fear-inducing situations, which contributes to the likelihood of developing PTSD or other anxiety disorders. Recent work strongly suggests that cigarette smoke can cause long-term disruption of the autonomic nervous system, likely as a result of the effects mentioned above on the HPA axis.

Animal studies have shown that nicotine caused extended fear responses, enhanced fear conditioning (22), delayed extinction of fear memories (23, 24), and disrupted safety learning (17, 25). In humans, children exposed to SHCS had higher rates of major depressive disorder and attention-deficit/hyperactivity disorder (20). A clear association has been observed between smoking and anxiety disorders, including PTSD (21). In emergency workers, smoking after exposure to a disaster was associated with increased PTSD symptoms; the authors suggest that smoking-related dysregulation of the hypothalamic-pituitary-adrenal axis contributes to increased PTSD symptoms (18).

The concept that nicotine exposure has long-term as well as short term effects on the autonomic nervous system, is distinct in concept from earlier literature. Previously the literature primarily documented the use of nicotine as a form of self-medication in individuals diagnosed with various affective disorders. The more recent accumulation of evidence for the negative effects of nicotine on the ability to recover from chronic stress and trauma, strongly support an intervention directed at the ANS of FA subjects to support recovery from the long-term health effects of SHCS exposure that occurred while working in a high-stress environment. Further, we think it likely that the disruption of ANS function consequent to SHCS exposure may exacerbate other morbidities associated with CS exposure, such as COPD and cardiovascular issues.

### Generalizability

This study addressed a specific population, flight attendants with workplace-related second-hand cigarette smoke exposure for more than 5 years. This, and the requirement to attend pre- and post-testing, limited the pool of eligible subjects to functionally mobile, current and former female FA over the age of 49. The generalizability of these results to the non-FA population—males, younger people, and those with more severe pathology—remains to be established; however, given the results of other MM studies with different populations, it appears likely that similar results would be obtained.

In comparing our study with other MM studies, we note that the intervention we developed differs from other MM practices in that it emphasizes integrating the principles of MM practice with daily life, rather than setting aside specific times for practice as is universally done in other studies (88). We believe that our approach improves effectiveness, compliance and accessibility, which are major hurdles for MM studies with a general population.

We have previously argued that precise and complete descriptions of MM interventions, used in research, need to be documented to support further studies of the impact of the intervention (46). These descriptions are also needed to form hypotheses relating to mechanism. Details of this intervention and the videos that we used to teach the MM protocol are available as Supplementary Materials. As with much clinical research, compliance remains an ongoing concern. We plan future studies to determine ways to increase compliance and effectiveness of MM training.

### Potential confounders

As noted above, baseline characteristics of the control group showed substantially better overall health than the intervention group. We do not believe this is likely to have distorted our results; if anything, we would speculate that participants in worse health would have been less likely to comply with the practices.

### Future directions

Future research needs to include further testing of refined training materials, modified methods of delivery, and the use of a larger cohort, to confirm the results from this study. We are currently exploring methods for making the videos more succinct and engaging, with the use of special effects, illustrative footage, images, sound effects, and music. Testing in other populations is also warranted but may also require specific modifications of the training for different populations. We believe that the specific style of intervention tested here offers considerable potential advantages over other forms, in that it can be effectively learned from videos, requires minimal or no separate practice time, and can be integrated with all the activities of daily life thus greatly increasing potential impact.

## Author contributions

PP, MC-G, and SF were responsible for project planning and execution. DZ provided testing resources. AB and PH provided video resources, video editing and project planning. TG contributed substantially to the manuscript. PP, MC-G, and CG were responsible for writing and major editing of manuscript. All provided editorial review.

### Conflict of interest statement

PP receives financial remuneration in his private practice for teaching material similar to that tested in this study. PP, MC-G, and CG receive compensation as consultants in areas related to the content of this study. The remaining authors declare that the research was conducted in the absence of any commercial or financial relationships that could be construed as a potential conflict of interest.

## Acronyms

**Table:**

6MWT	Six minute walk test	A standard test for assessing functional ability, the distance walked in 6 min
AD	Autonomic dysfunction	
ANS	Autonomic nervous system	Portion of the nervous system controlling those physiological functions outside normal voluntary control.
BP	Blood pressure	
CS	Cigarette smoke	
COPD	Chronic obstructive pulmonary disease	Chronic disease involving obstruction of the small airways and alveolar tissue disruption
hs-CRP	High sensitivity C-reactive protein	Bio-marker of systemic inflammation
DHEAS	Dehydroepiadrosterone sulfate	A steroid hormone precursor
DVD	Digital video disc	Standard vehicle for presenting video
FA	Flight attendants	
FAMRI	Flight attendant medical research institute	The foremost organization promoting research into medical problems affecting flight attendants.
FEF	Forced expiratory flow	Spirometric measurement, flow of air during forced exhalation
FEV1	Forced expiratory volume one	A measure of lung function: the maximum volume of air breathed out in a forcible exhalation in 1 s
FVC	Forced vital capacity	A measure of lung function: the total volume of air breathed out in a complete forcible exhalation
GOLD	Global initiative on Obstructive Lung Disease	A research and information group that is the principal global authority on the nature and treatment of COPD
HR	Heart rate	
MAIA	Multidimensional assessment of interoceptive awareness	A questionnaire designed to evaluate several dimensions of interoceptive awareness
MM	Meditative movement	A newly identified form of exercise characterized by a meditative state of mind, deep relaxation, movement, and attention to the breathing. Qigong and Yoga are the best known examples
PEF	Peak Expiratory Flow	The maximum rate of flow achieved in a forced expiration
PI	Principal investigator	
RCT	Randomized controlled trial	The premier form of research study in which participants are randomly assigned to control or intervention group for the purpose of objective assessment of an intervention
SHCS	Second-hand cigarette smoke	Cigarette smoke inhaled by someone other than the smoker; ambient smoke not inhaled directly through the cigarette
